# First person – Chiara Herzog and David Greenald

**DOI:** 10.1242/bio.052654

**Published:** 2020-05-04

## Abstract

First Person is a series of interviews with the first authors of a selection of papers published in Biology Open, helping early-career researchers promote themselves alongside their papers. Chiara Herzog and David Greenald are co-first authors on ‘RNA-seq analysis and compound screening highlight multiple signalling pathways regulating secondary cell death after acute CNS injury in vivo’, published in BiO. Chiara is associate business development manager at BioClavis Ltd, Queen Elizabeth University Hospital, Teaching & Learning Centre, Glasgow, working towards making a difference for patients and advancing medicine and diagnostics with innovative technologies. David is a senior research technician in the lab of Dr Rod Mitchell at the Centre for Reproductive Health, The Queen's Medical Research Institute, Edinburgh, investigating small compound screens and how they can be beneficial for academic progress as well as medically relevant.


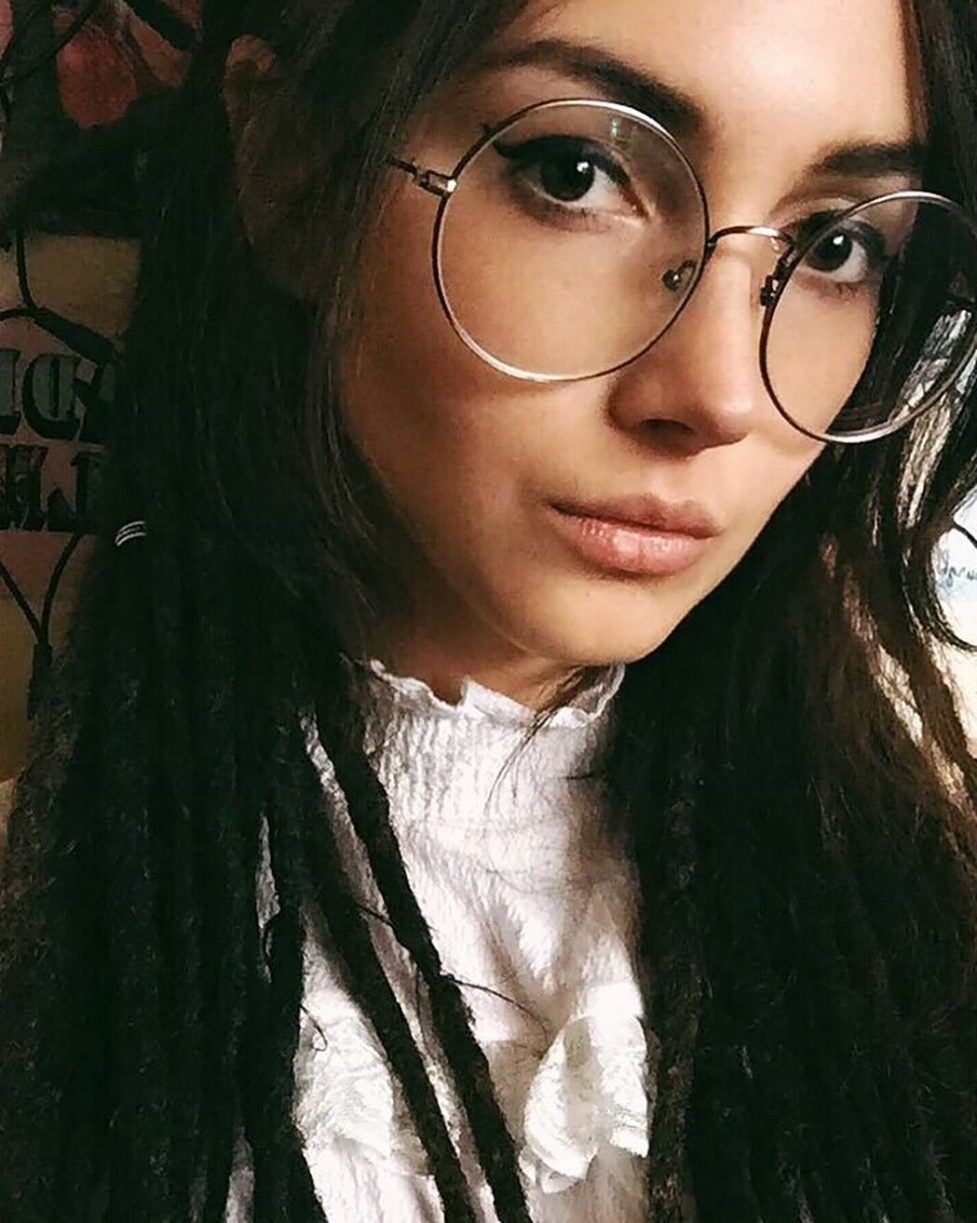


**Chiara Herzog**

**What is your scientific background and the general focus of your lab?**

C.H.: My background is originally in molecular medicine at the intersection of patient and laboratory research, but my fascination with the brain pulled me towards a specialisation in neuroscience. Our lab focused on elucidating molecular mechanisms leading to successful neuronal repair in zebrafish with the hope that ultimately these findings could be applied to humans. In contrast to humans, zebrafish are intrinsically capable of recovering from a variety of injuries, including traumatic brain injuries. During my PhD, I became particularly interested in how the immune system can regulate repair and regeneration in the brain. While it was becoming clear that the immune system, in particular macrophages, plays a key role in orchestration of successful wound repair in many other tissues, the role of these cells in the brain was less clear.

D.G.: I started out as a neuroscientist, with a particular interest in motor neuron disease, where I did projects that used both mouse and zebrafish models of disease. Working with zebrafish as a model organism opened my eyes to how versatile zebrafish are as an animal model, and I decided to pursue a PhD where I looked at chemical and genetic modulators of the hypoxia pathway. This project involved screening for drugs that modulated the hypoxia pathway and characterising the hit compound in terms of its transcriptional response. I decided to return to neuroscience to work in the lab of Leah Herrgen, where I worked to understand the molecular mechanisms, key cellular actors and early signalling pathways that coordinate neuronal repair following traumatic brain injury (TBI). My project focused on finding compounds that modulate repair, in the hope that this would provide a starting point for developing drugs for therapeutic intervention following TBI.

**How would you explain the main findings of your paper to non-scientific family and friends?**

Traumatic brain injuries are one of the leading causes of death and disability worldwide, particularly in low- and middle-income countries. It is clear that we need a better understanding of what is happening in the brain after an injury: while researchers have been trying to improve the recovery of patients from brain injury with a variety of drugs for decades, no clinical trials to date have demonstrated an improvement in patient outcome. In our study, we used several different approaches to tease apart the molecular cascades occurring in the brain after an injury in a species that is, contrary to humans, capable of repairing its brain after an injury. Not surprisingly, we find that not one, but several different signalling pathways are active. Importantly, looking at cells of the immune system, we find that they respond with an upregulation of genes that result in improved recovery.

“… we used several different approaches to tease apart the molecular cascades occurring in the brain after an injury in a species that is, contrary to humans, capable of repairing its brain after an injury.”

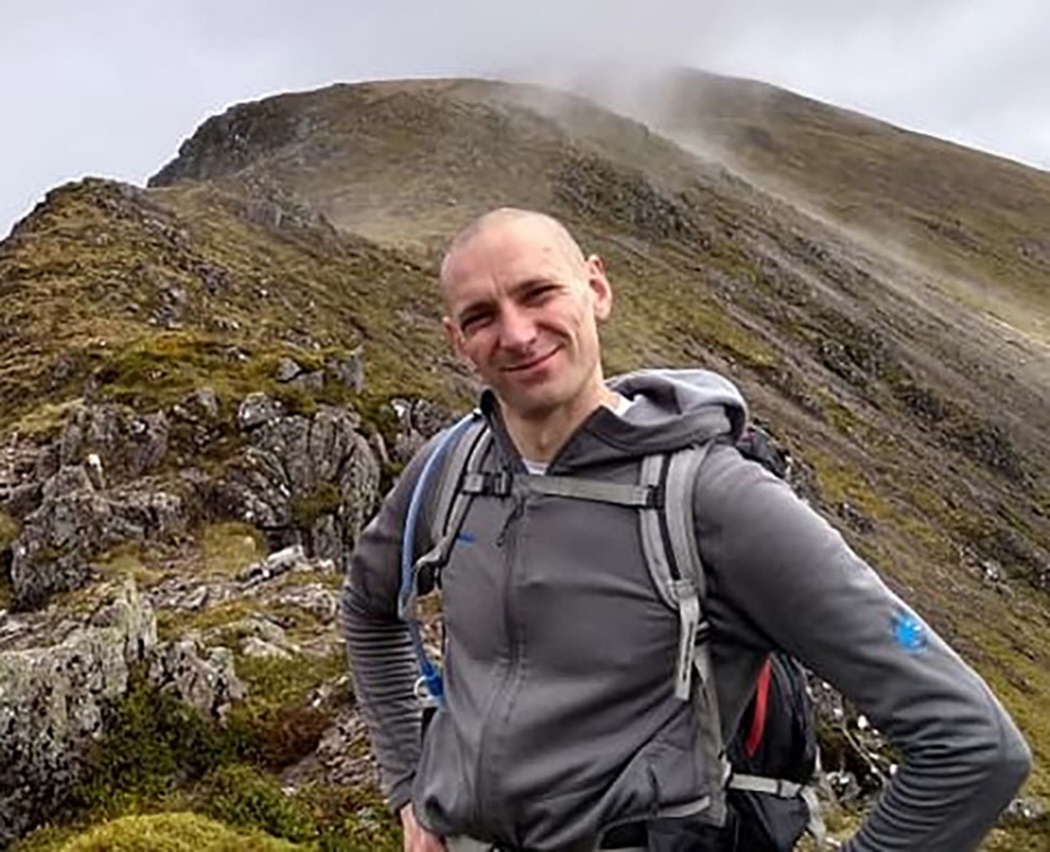


**David Greenald**

**What are the potential implications of these results for your field of research?**

C.H.: Our findings highlight that it's not enough to look at one specific pathway when trying to identify factors promoting regeneration or recovery. After an injury in the brain, there is lots going on; targeting one pathway may influence others in a negative way. One of the main findings is that microglia upregulate genes that are associated with reduced secondary cell death. It would be interesting to compare these findings to what we see in mammalian species, and see what happens when “protective” transcripts are enhanced.

D.G.: Further to this, we show that pharmaceutical intervention after traumatic brain injury is able to decrease the amount of secondary cell death. This acts as a proof of principle that our injury paradigm can be useful for discovering drugs that could be used to treat head injuries.

“…we show that pharmaceutical intervention after traumatic brain injury is able to decrease the amount of secondary cell death.”

**What has surprised you the most while conducting your research?**

The regenerative capacity of the zebrafish brain – it's really amazing!

**What, in your opinion, are some of the greatest achievements in your field and how has this influenced your research?**

C.H.: I think there has been a lot of progress in the field of neuroimmunology, and it is now generally accepted that microglia carry out key functions during development, homeostasis and repair. This has been enabled by new tools like in vivo imaging, (single- cell) RNA-Seq, etc., and model organisms.

D.G.: In my relatively short career I've seen huge advances in using zebrafish for *in vivo* imaging. The development of real-time fluorescent probes such as GCaMP, which are used as transgenic reporter lines, and the ease with which mutants can be made using CRISPR gene editing, combined with the advances in and availability of top-end microscopes such as spinning disk and light-sheet technologies, mean that the ease with which complex biological phenomena can be visualised and manipulated has grown exponentially.

**Figure:**
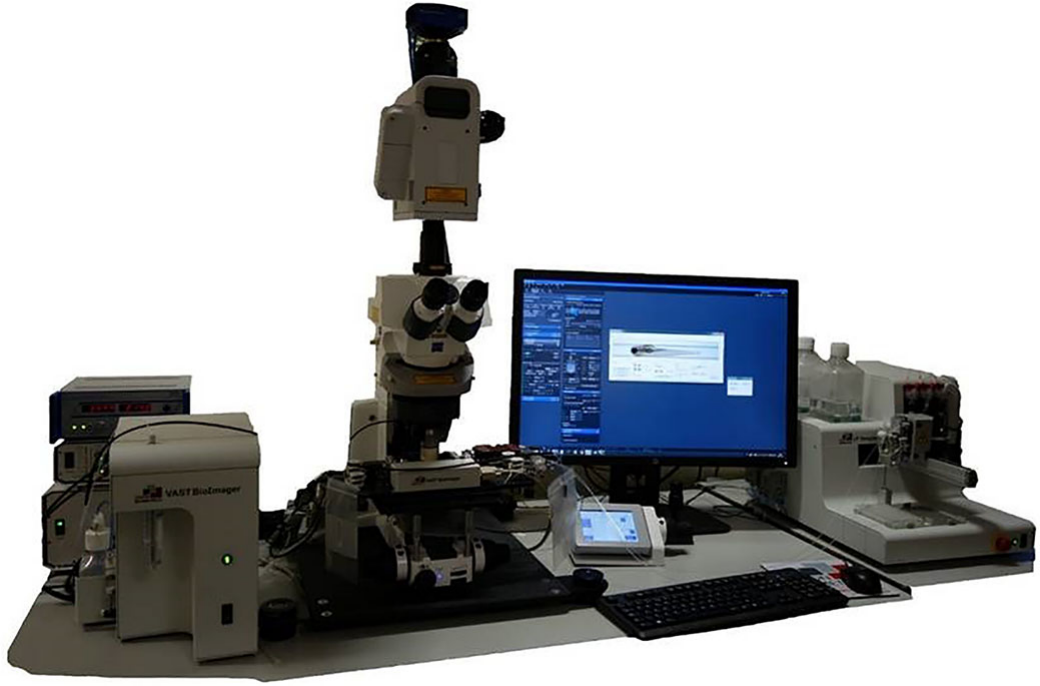
**A picture of the VAST Bioimager, which we used for our high-content drug screen in zebrafish larvae.** It is an automated robotic imaging platform coupled to a spinning disk fluorescence microscope. It removes the need to manually embed each individual fish in agarose by retrieving, aligning and imaging each larva automatically, allowing for a much higher number of larvae to be imaged per day than previously possible (we screened 200 fish per day).This is a complete game-changer in the field of zebrafish drug screening as it allowed us to look at a complex phenotype in detail and en masse.

**What changes do you think could improve the professional lives of early-career scientists?**

C.H.: The life of an early-career scientist is extremely dynamic, and not always in a good way. It's great to learn something new every day, but when you are constantly having to worry about what your next career step will be, this can decrease the joy of discovery for many. Contracts are often too short to plan meaningful long-term experiments and the frequent need to relocate can put a strain on social life and mental health. Although I don't think there will be a perfect solution, I believe providing more stability for early-career researchers will be an important topic to tackle in the next decade.

D.G.: Better pay, greater job security and improved working conditions. From my experience, the majority of scientists enjoy their work and find it fulfilling, they are willing to make sacrifices to achieve the goals of their projects, but many leave academia not because they have not been successful or because they lack talent but because they are overworked, suffering from stress, and need better job security to raise a family.

**What's next for you?**

C.H.: I have moved on to a position in a spin-out specialising in molecular diagnostics for precision medicine and translational research using a proprietary high-throughput transcriptomics platform. Although I have left the bench, I am still following the latest research in a wide range of fields, also outside my position.

D.G.: I have moved on to a similar position in a different field researching the human testis in relation to fertility preservation, disorders of sexual development and testicular cancer.
